# Supplementary material for: Enabling Clonal Analyses of Yeast in Outer Space by Encapsulation and Desiccation in Hollow Microparticles
Source: Life (Basel). 2022 Jul 31;12(8):1168. doi: 10.3390/life12081168 (PMC9410522; doi:10.3390/life12081168)
Supplement: Supplementary file 1 [file life-12-01168-s001.zip › life-1803511_Supplementary_Material_REV1.pdf]

Supplementary Material

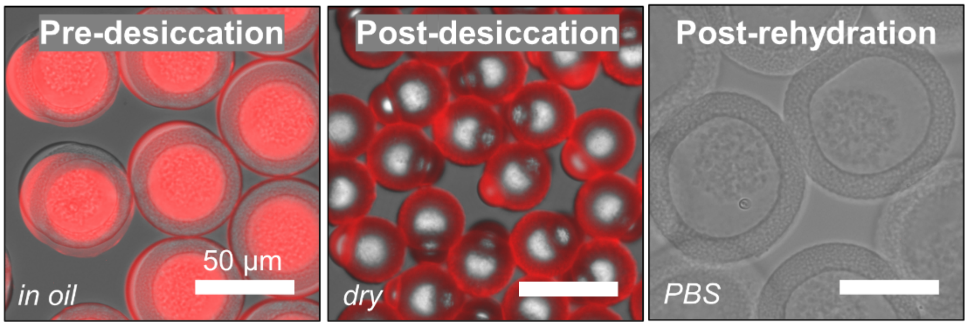

Figure S1. Dextran washes out of particles upon rehydration. Fluorescently labeled dextran (red) was encapsulated in particles and imaged through the desiccation process. Before rehydration, when particles were either in oil pre-desiccation or dry post-desiccation, the fluorescent dextran was associated with the particles. After rehydration with PBS, no fluorescent dextran can be seen, indicating that it diffused out of the particle as expected. The dextran phase was composed of unlabeled 9-11 kDa dextran at 199.1 mg/mL and 9-11 kDa rhodamine-dextran at 0.9 mg/mL, all in PBS pH 6.1. All images were taken at 40x magnification on an EVOS FL Cell Imaging Microscope with RFP and brightfield overlay; the pre-desiccation and post-rehydration images were taken with 100% RFP illumination and 250 ms exposure. The post-desiccation image was taken with 90% RFP illumination and 30 ms exposure to avoid camera saturation. These particles were vacuum dried for 24 hours and rehydrated immediately.

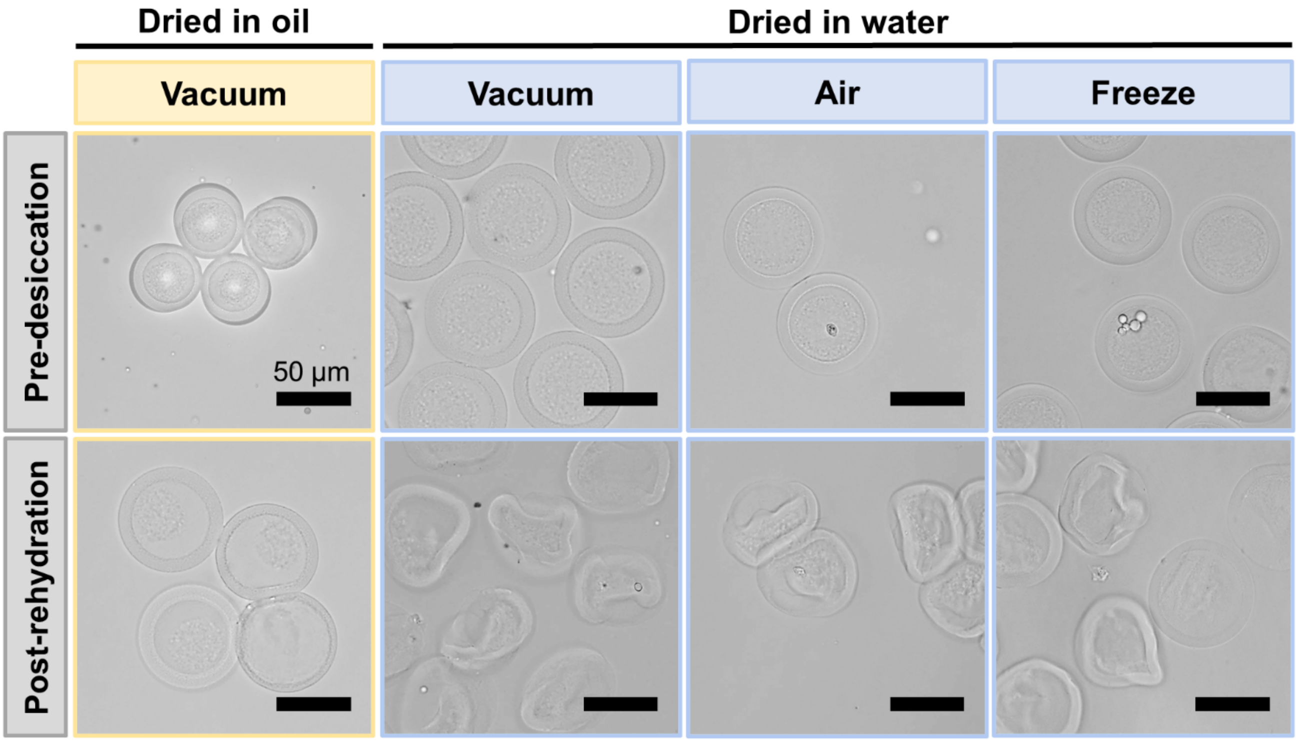

Figure S2. Desiccation in Novec oil yields better morphology after rehydration than desiccation in water. Particles vacuum dried in Novec oil with 0.5% Pico-Surf (leftmost column) possess a distinct core-shell morphology after rehydration in YPD medium. In contrast, particles phase transferred to water before desiccation are collapsed and shriveled after rehydration. Both sets of vacuum dried particles were fabricated in the same batch, vacuum dried for 22 hours, and rehydrated immediately. Air and freeze dried particles were fabricated in a separate batch, desiccated according to methods in **2.4 Desiccation, storage, and rehydration**, and stored for 7 weeks before rehydration.

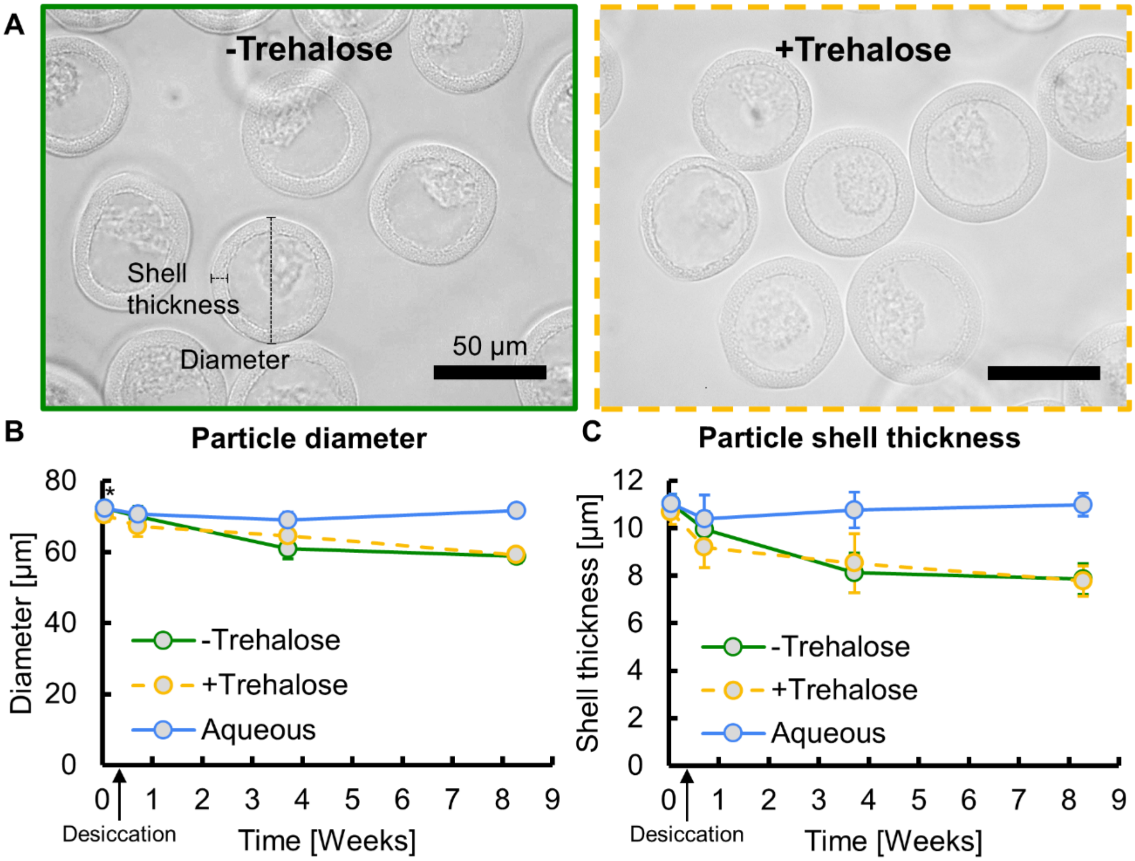

Figure S3. +Trehalose and -Trehalose PicoShells have similar morphology. (**A**) Rehydrated -Trehalose and +Trehalose particles look similar 3.5 weeks after desiccation. (**B**) The diameters of -Trehalose and +Trehalose particles are only significantly different at 0 weeks (0 weeks, * $p < 0.05$; 0.5 weeks, $p = 0.071$; 3.5 weeks, $p = 0.052$; 8.5 weeks, $p = 0.90$). (**C**) The shell thickness of -Trehalose and +Trehalose particles are not significantly different at any timepoint (0 weeks, $p = 0.17$; 0.5 weeks, $p = 0.16$; 3.5 weeks, $p = 0.71$; 8.5 weeks, $p = 0.97$). *rad51***Δ** particles are not compared here because they only differ from +Trehalose particles by encapsulating *rad51***Δ** yeast instead of wild type yeast. Note that the 0-week timepoint for +Trehalose represents particles that were phase transferred into aqueous phase instead of being desiccated; the 0.5-week timepoint is the first post-desiccation timepoint. Also note that at the 0-week pre-desiccation timepoint, -Trehalose particles are the same condition as Aqueous particles and so the data is the same. Data are plotted as a line plot showing the mean and one s.d. (n = 5-11 particles).

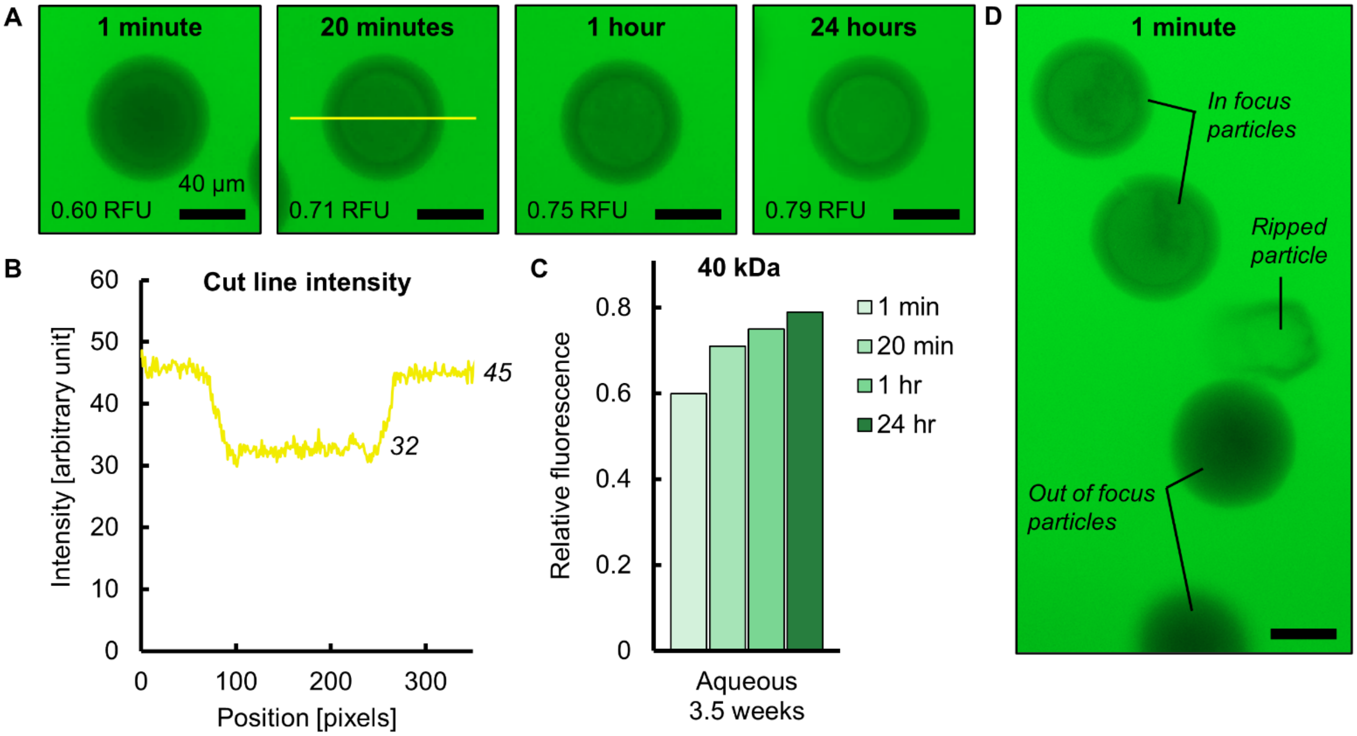

Figure S4. Diffusion measurements over 24 hours. (**A**) Particles were imaged in FITC-dextran solution at four timepoints – 1 minute, 20 minutes, 1 hour, and 24 hours. As FITC-dextran diffuses into the particle, the intensity inside the particle approaches the intensity outside the particle. To obtain relative fluorescence measurements, cut lines (yellow) were taken across the particle and the background on either side (see *20 minutes*). (**B**) The intensity outside and inside the particle were averaged (for *20 minutes*, 45 units outside the particle and 32 units inside the particle) and taken in a ratio to give a relative fluorescence unit (32/45 ≈ 0.71 RFU). (**C**) The relative fluorescence of these aqueous particles, incubated with 40 kDa FITC-dextran after 3.5 weeks of storage in PBS, gives a 24-hour diffusion profile as 40 kDa FITC-dextran diffuses into the particle. Data are plotted as a bar plot showing individual particle RFU measurements. (**D**) Multiple images were taken of each particle set to measure each particle when it is in focus. Ripped particles are rare, fill immediately with FITC-dextran solution, and were excluded from analysis. This image is of +Trehalose PicoShells under the same conditions as (**A**).

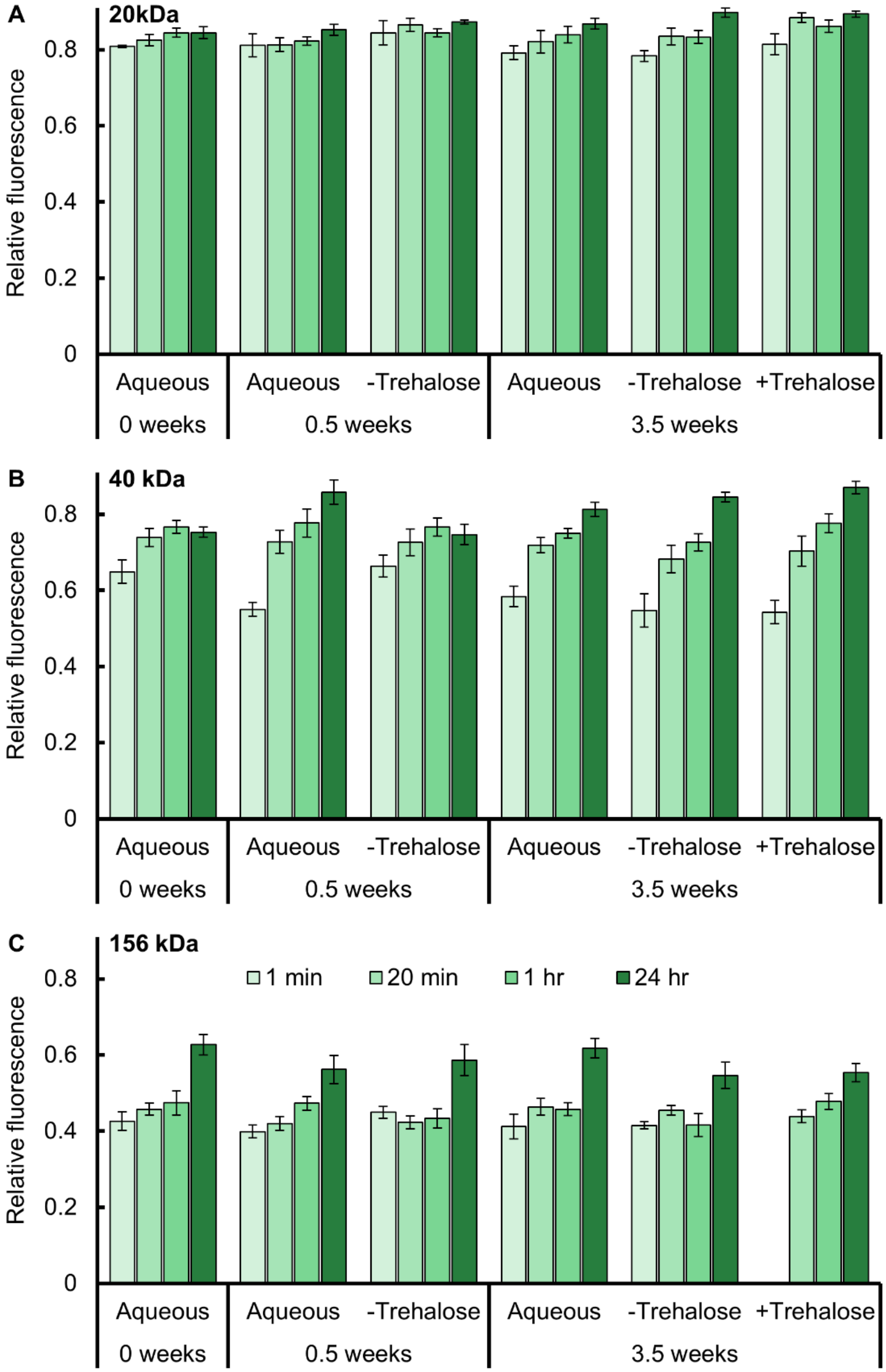

Figure S5. Diffusion profiles of aqueous and rehydrated particles. Three different sized FITC-dextran molecules – (**A**) 20 kDa, (**B**) 40 kDa, and (**C**) 156 kDa – were assayed for diffusion into aqueous PicoShells (never dehydrated), and rehydrated -Trehalose and +Trehalose PicoShells. The four bars for each condition represent the relative fluorescence after 1 minute, 20 minutes, 1 hour, and 24 hours. The aqueous and -Trehalose particles come from the same particle fabrication batch, so the Aqueous 0 weeks condition also represents the -Trehalose 0 weeks no-desiccation condition. The 1-minute timepoint for 156 kDa +Trehalose was not captured. Data are plotted as a bar plot showing the mean and one s.d. (n = 3-12 particles).

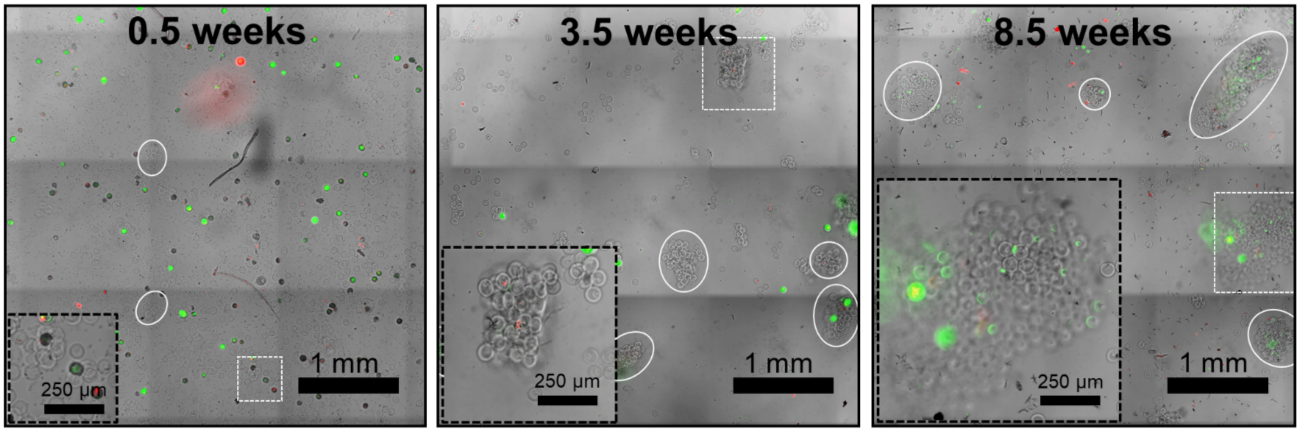

Figure S6. Increasing particle aggregation with storage duration. The white ellipses and dashed boxes mark particle aggregates. The white dashed boxes mark the insets. When rehydrating 0.5 weeks after desiccation, there are few, small aggregates. By 3.5 weeks, there are more, larger aggregates, and by 8.5 weeks, some aggregates span focal planes. These images are stitched tile images from the viability and growth measurements with the well edges cropped out. The particles are +Trehalose, vacuum dried in Novec oil and Pico-Surf for 72 hours.

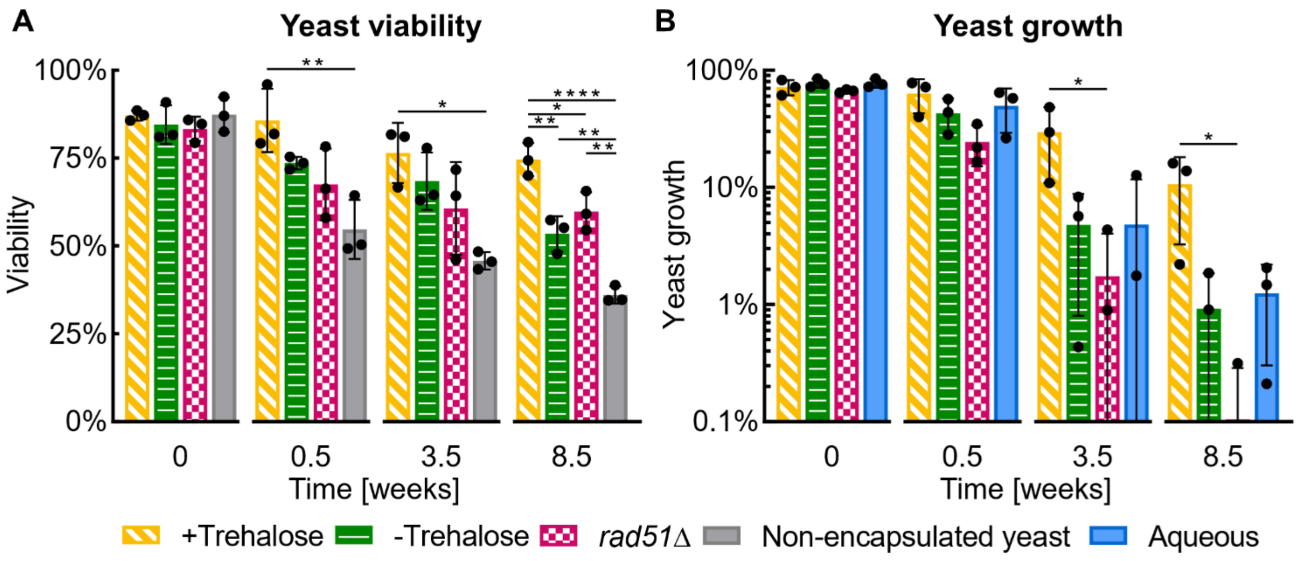

Figure S7. Raw, non-normalized yeast health. Yeast health was quantified via (**A**) viability and (**B**) growth potential at four timepoints. Timepoint 0 weeks shows the viability and growth of PicoShells that were phase transferred into PBS shortly after particle fabrication and assayed immediately, never desiccated. Timepoints 0.5, 3.5, and 8.5 weeks show the viability and growth of PicoShells that were desiccated and stored for the specified time before rehydration and assaying. Yeast growth shows the portion of the population that grew to fill or swell the PicoShell after incubation for 16 hours. Where fewer than three datapoints are shown, the unmarked datapoints had no growing yeast in any particles. Data are plotted as bars showing the mean and one s.d. as well as biological replicate datapoints (n = 3 wells). Significance markers are shown only within each timepoint. * $p \leq 0.05$, ** $p \leq 0.01$, **** $p \leq 0.0001$
